# Predicting scheduled hospital attendance with artificial intelligence

**DOI:** 10.1038/s41746-019-0103-3

**Published:** 2019-04-12

**Authors:** Amy Nelson, Daniel Herron, Geraint Rees, Parashkev Nachev

**Affiliations:** 1 Institute of Neurology, UCL, London, WC1N 3BG UK; 2 NIHR UCLH Biomedical Research Centre, Research & Development, Maple House Suite A 1st Floor, 149 Tottenham Court Road, London, W1T 7DN UK; 3 Institute of Cognitive Neuroscience, UCL, London, WC1N 3AR UK; 4 Faculty of Life Sciences, UCL, London, WC1E 6BT UK; 5 Wellcome Trust Centre for Neuroimaging, UCL, London, WC1N 3BG UK

**Keywords:** Health policy, Health care economics, Magnetic resonance imaging

## Abstract

Failure to attend scheduled hospital appointments disrupts clinical management and consumes resource estimated at £1 billion annually in the United Kingdom National Health Service alone. Accurate stratification of absence risk can maximize the yield of preventative interventions. The wide multiplicity of potential causes, and the poor performance of systems based on simple, linear, low-dimensional models, suggests complex predictive models of attendance are needed. Here, we quantify the effect of using complex, non-linear, high-dimensional models enabled by machine learning. Models systematically varying in complexity based on logistic regression, support vector machines, random forests, AdaBoost, or gradient boosting machines were trained and evaluated on an unselected set of 22,318 consecutive scheduled magnetic resonance imaging appointments at two UCL hospitals. High-dimensional Gradient Boosting Machine-based models achieved the best performance reported in the literature, exhibiting an area under the receiver operating characteristic curve of 0.852 and average precision of 0.511. Optimal predictive performance required 81 variables. Simulations showed net potential benefit across a wide range of attendance characteristics, peaking at £3.15 per appointment at current prevalence and call efficiency. Optimal attendance prediction requires more complex models than have hitherto been applied in the field, reflecting the complex interplay of patient, environmental, and operational causal factors. Far from an exotic luxury, high-dimensional models based on machine learning are likely essential to optimal scheduling amongst other operational aspects of hospital care. High predictive performance is achievable with data from a single institution, obviating the need for aggregating large-scale sensitive data across governance boundaries.

## Introduction

Failure to attend hospital appointments needlessly delays clinical care and consumes resource better spent on improving its quality.^1^ Its reach is global: the African continent (43.0%), South America (27.8%), Asia (25.1%), North America (23.5%), and the rest of Europe (19.3%).^2^

That attendance rates have remained relatively unchanged over the past 10 years suggests the problem is anything but simple.^1^ Two interacting factors arguably account for its difficulty. First, the comparative infrequency of non-attendances means any intervention applied indiscriminately to all patients—such as blanket phone call reminding—is wasted on the majority of its recipients, rendering further escalation inefficient. Second, systems that target interventions by predicting individual non-attendance are difficult to devise because the diversity of probable causes—ranging from behavioral predispositions to environmental events—is too wide. The temptation is to discard all but the most generic predictive features, relying on simple, linear, *low-dimensional* statistical models. For example, of the eight studies to quantify out-of-sample attendance prediction performance identified in a systematic review of the literature (see Supplementary Information), only three used non-linear models, and none included more than 49 variables (Table 1). But the mathematical framework behind such models is designed to make simple *inferences* about groups, not complex *predictions* about individuals. Simple models, chosen for their intelligibility and generalizability, are ill-suited to predicting individual events where the causal field is wide.

**Table 1:** Summary of all published models of scheduled appointment attendance in healthcare—ranked by area under the receiver operating characteristic curve in order of performance—for which out-of-sample metrics are available

Model	Type	Variable count	Predictive performance (AUC)
Stacking^17^	Non-linear	18	0.846
XGBoost^5^	Non-linear	42	0.834
Neural network^6^	Non-linear	Not available	0.81
Logistic regression^16^	Linear	38	0.75
Logistic regression^7^	Linear	49	0.713
Logistic regression^17^	Linear	14	0.706
Sums of exponentials for regression^8^	Linear	17	0.706
Logistic regression^9^	Linear	13	0.702

Note: More complex, high-dimensional models tend to exhibit greater predictive power

There is another way. The complexity of a mathematical model—its ability to absorb non-linear associations and complex interactions between many variables—is limited only by the availability of data and the scale of the computational resource applied to it. Combining machine learning with large-scale data allows us to create rich, complex, *high-dimensional* models able to operate within wider causal fields. If such models perform and generalize better than simpler variants their one defect—lack of easy intelligibility—is far outweighed.

Complex models may not only predict attendance, enabling targeted intervention, but also *prescribe* it by matching detailed appointment and patient characteristics. By capturing individual variability better, they may also be used to infer systemic, modifiable hospital causes of non-attendance currently obscured by the many other factors in play. Complex models both potentially enhance existing interventions and open the way to implementing categorially new ones.

Across most healthcare systems, capacity limitations distribute non-urgent initial secondary care appointments across a wide interval—18 weeks in the UK National Health Service (NHS)—where patients have varying freedom over the choice of an appointment slot; subsequent appointments are distributed even more broadly as clinical needs dictate. Scheduling is communicated by mail, sometimes confirmed by telephone, text, or email reminders. The greater resource complexity of secondary care amplifies the cost of each missed appointment, accumulating, in the UK alone, an estimated £1 billion annual loss for secondary care on a ~8.5% non-attendance rate compared with £150 million for primary care on a 7.9% non-attendance rate.^3,4^

Here, we focus on an important exemplar of hospital outpatient scheduling: magnetic resonance imaging (MRI). The breadth of coverage across multiple medical domains, the diagnostic weight of the investigational class, and the high, fixed unit cost here combine plausible generalizability with a substantial margin of potential benefit from improved attendance rates.

Studying a large sample of MRI appointments across two large UK hospitals, we sought to answer two related questions: what is the relationship between the complexity of predictive models of attendance and their predictive performance, and can sufficient predictive performance be achieved to render targeting cost-effective? If complex models are convincingly shown to be required for optimal performance, a reorientation of hospital scheduling analytics to machine learning-based modelling would be indicated; if there is no difference between simple and complex approaches, then other avenues for improving scheduling ought to be pursued. We further propose a framework for evaluating such models that takes into account the relative cost of non-attendance and the effort of preventing it.

## Results

### Data distribution

Summary analysis revealed a typical overall attendance distribution, and a wide diversity of MR imaging types across the set of 22,318 appointments (see Supplementary Figs 1 and 2). Demographic and other clinical details are not available on our radiology scheduling system, and were not accessible to us within the operational optimization remit of our study.

### Performance

**Fig. 1:**
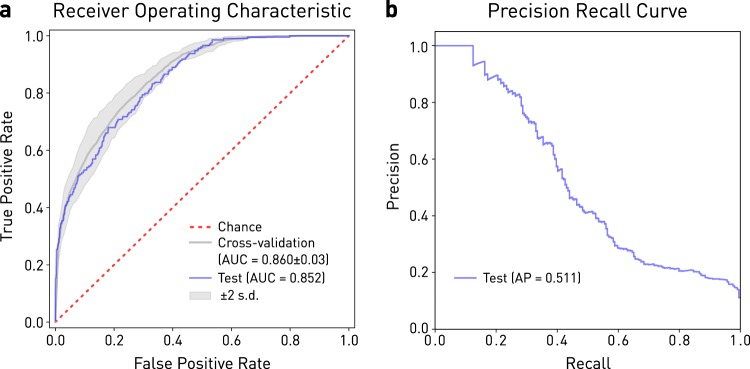
Performance of the optimal model based on gradient boosting machines incorporating 81 variables. **a** Receiver Operating Characteristic curve for performance on the held-out test set (blue line, AUC = 0.852), on cross-validation (mean = thick gray line, AUC = 0.860, two standard deviations (s.d.) = thin gray lines, ±0.03), and chance (red dotted line). **b** Precision-Recall curve on the held-out test set, yielding an Average Precision (AP) score of 0.511

The top performing model—based on GBM with 81 features—achieved an AUC of 0.852 and an average precision of 0.511 on the out-of-sample test set (Fig. 1). The training time for this model was 16.6 s. Test set AUC was faithful to the mean training AUC obtained by 6-fold cross-validation (0.860 ± 0.01 sd).

### Model complexity and optimal variable number

**Fig. 2:**
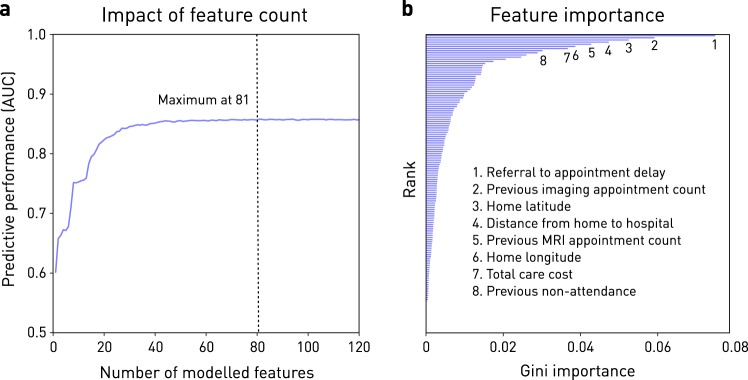
The impact of model dimensionality. **a** Performance on the held-out test set across Gradient Boosting Machine-based models incorporating features recursively eliminated in order of Gini-importance from the full model. Note that full performance is reached only after the inclusion of 81 features. **b** Gini-importance based ranking of the features in the best Gradient Boosting Machine model; the top 8 are labelled. Note the wide distribution of feature importance across variables

For the top performing model architecture, predictive performance increased with the addition of further variables up to 81 (Fig. 2a). Escalating dimensionality did not incur prohibitive computational penalties: training times with 20, 30, and 81 variables were 7.1, 8.9, and 16.6 s respectively. The distribution of Gini-importance feature weighting was broad (Fig. 2b). Summary performance increased with the expressive capacity of the evaluated architectures: logistic regression, SVM, Random Forest, and AdaBoost models achieved cross-validation AUCs of 0.771, 0.792, 0.826, and 0.848, with training times of 3 min 23 s, 5 min 27 s, 8.3 s, and 9.1 s, respectively. Plots of the minimal effects of class weighting, random under-sampling, and SMOTE oversampling on model performance are available in Supplementary Fig. 3.

### Impact

**Fig. 3:**
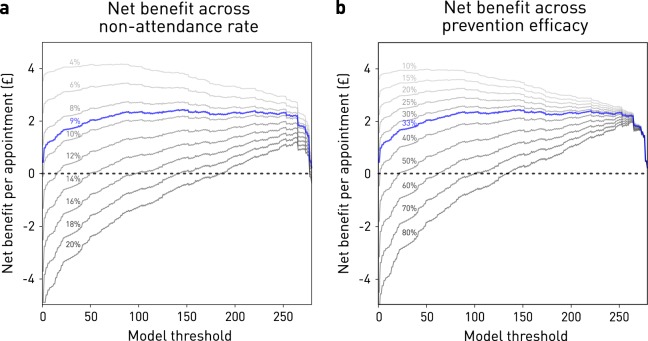
Net benefit simulations with the optimal model. **a** Estimated net benefit per attendance in pounds sterling as a function of the chosen model threshold—the output model value at which the attendance class is assigned—in blue at the 9% non-attendance rate in our dataset, and in shades of gray at increments between 4 and 20%. Net benefit falls with reduced attendance, but there is always a model threshold at which it is positive. **b** Estimated net benefit per attendance in pounds sterling as a function of the chosen model threshold, in blue at the 33% estimated mean intervention efficacy, and in shades of gray at increments between 10 and 80%. Net benefit falls with increased efficacy, but there is always a model threshold at which it is positive

Call efficiency for our best model was 0.19, equating to a number-needed-to-call of 5.3, set at a test threshold corresponding to 90% sensitivity and 41% specificity. This is more than double the baseline of 0.09 and 11, respectively. The operating net benefit of using the model over intervening in all patients peaked at £3.15 per appointment, but remained positive over a wide range of non-attendance prevalences and intervention efficacies (Fig. 3). Given an estimated capital cost for infrastructure and development of ~£20,000, this yields a break-even point of ~6350 scheduled appointments. If the observed performance is confined solely to the ~20,000 MRI outpatient appointments booked annually at the average NHS hospital trust, the break-even point would be reached after ~83 working days. If the observed net benefit performance is replicated across the mean ~800,000 out-patient appointments annually in the average NHS hospital trust, the break-even point would be reached within a few days. Naturally, equivalent predictive fidelity may not be achievable outside our specific domain, and the benefit of prevented non-attendances will vary with the nature of the appointment, but these estimates can accommodate a wide margin of error.

## Discussion

Our analysis of a large, diverse, unselected set of consecutive magnetic resonance radiological scheduled appointments demonstrates that predicting attendance demands high-dimensional, high-capacity modelling. Indeed, our optimal model is both the most complex and the best performing in the published literature.

Hospital attendance is bound to be a complex target of prediction given the wide field of plausibly material factors. Behavioral predispositions, physical constraints, clinical manifestations, hospital service characteristics, geography, transport, and weather will all interact in complex ways to determine the outcome of any particular appointment. Some factors consistently carry more predictive information than others, both in our study (where examined) and the wider literature: non-attendance history,^5–9^ referral-to-appointment time,^5–7,10^ appointment day and month,^6,8,10^ age,^5–8^ ethnicity,^5,8^ and weather.^6,10^ Factors identified here but not yet comprehensively examined elsewhere include patient home latitude and longitude, distance from home to hospital, and the total cost of patient activity. Our primary task, however, is not to identify the most strongly predictive factors but to identify a modelling approach that yields the best predictive performance overall. A great deal of information may be distributed across a wide field of weakly predictive factors: the right modelling architecture could harness this to achieve much better performance than analysis of each factor in isolation or linear combination would suggest. Indeed, that the best performance in our study was achieved by the most complex models indicates exploration of even greater complexity is likely to be rewarding.

It is plausible that our analysis does not set a ceiling on maximal performance since demographic information—of reported importance in almost all previous models^5–9^—is unlikely to have been rendered wholly redundant by the field of modelled covariates. We did not include demographics because our radiology administrative system—in common with many others—does not capture them, hindering the real-world implementation of models that require them. Equally, even greater performance might be achievable with architectures of greater expressive power—such as those based on artificial neural networks—but at the cost of potentially inhibitory complexity of development and optimization.

Though limited in their actionable antecedence, fluctuations in transport and weather should provide predictive information more weakly supplied by geography and season. Richer parameterization of the patient’s clinical background should also sharpen the contribution of the clinical context, in the present models conveyed solely by the type of scheduled investigation.

Equally, the observed performance is unlikely to be limited to our particular dataset, for five reasons. First, performance was quantified not by model statistics, but on out-of-sample data wholly unseen by the model during training and optimization. This differs from prospective testing only in that the data already existed, which does not materially alter the statistical rigor of the test. Second, our dataset is diverse, unselected, and consecutively accumulated over a broad interval, so likely representative of data of this kind. Third, though complex, our models incorporate a number of features that are small in proportion to the size of the dataset, limiting the risk of overfitting. This is reflected in the stability of model training, the minimal discrepancy between cross-validation and held-out test performance, and the broad agreement between architectures of comparable expressive power. Fourth, the nature and rank of the most predictive features are both in keeping with prior expectations and dominated by general features of appointments. Fifth, by choosing a specialist radiological modality we can both cover a wide diversity of clinical conditions and achieve better sampling of relatively narrow contexts that nonetheless aggregate to a substantial proportion of healthcare activity.

Special treatment must be given of the question of model generalization to other institutions and clinical domains. At another institution, the weighting of factors may well be different, reflecting different populations and operational procedures; in another clinical domain, wholly different factors may arise. Where a model is optimally fitted to a particular attendance task, it should *not* perform as well elsewhere; if it does, then its fit is likely to have left too much room for improvement. Our sole concern here is predictive fidelity—naturally sustained over time—for a particular institution and a particular clinical domain.

Of course, given sufficient data, a more complex model could learn to absorb such factors together with all others. But given the ubiquity of attendance data at most hospitals—projected deep into the past—there are no practical obstacles to creating bespoke models, or at least retraining models heavily on local data. Indeed, single-site models are desirable owing to the information governance obstacles to pooling sensitive data across institutions. We do not need model generalizability, only replicability of the high-dimensional modelling approach.

Predicting attendance does not, in itself, prevent it, so the impact of better prediction depends on the efficacy and relative cost of an intervention, contextualized by attendance rate. The relative cost of a telephone call (~£6) and a missed appointment typical of complex radiology (~£150) leaves room for substantially narrower margins, even at relatively low interventional efficacies, given the former is reasonably uniform across the industry, whereas the latter may be substantially lower. Our focus on telephone calls here is justified by the loss of penetration of fully-automated means of reminding—text messages, for example—caused by the rapid proliferation of different mobile messaging applications.

Our models of net benefit encompass a much wider range of intervention efficacies than is reported in the literature: 33–39%.^11–13^ That we observed a positive net benefit across our modelled range suggests real-world variations in this parameter are unlikely to limit the utility of the approach. Equally, the net benefit remains positive across the full range of realistic attendance rates (Fig. 3). Accumulated across the large number of scheduled events at an average healthcare institution in the UK— ~20,000 MR scans and ~800,000 outpatient appointments annually—the benefit is plausibly large enough to justify pursuing even relatively small improvements in predictive performance.

Targeted reminding is only one way of using high-dimensional models to improve attendance. Information available at the time of booking may be used not only to predict attendance but to *prescribe* the appointment characteristics most likely to deliver it. While the nature of the appointment is clinically determined, its timing and transport mechanisms are free to vary. Collaborative filtering algorithms can be deployed here to match multiple characteristics of the patient and the appointment, reducing the risk of non-attendance at the time of scheduling.^14^ Second, a comprehensive characterization of the factors impinging on attendance enhances our ability to identify a subset—either of patients, such as transport means, or the institution, such as clinic times—that can be systemically modified.^15^ Such inference is essential to optimizing the operational framework of scheduled healthcare activity.

We have achieved excellent predictive performance with models trained only on routinely collected administrative data, built with open-source tools, and estimated and validated on conventional hardware. Though more complex modelling, especially involving dynamic, external factors, may require more complex systems, effective implementations are likely to be economical. Note that the computational cost of escalating dimensionality and expressive capacity is relatively modest in proportion to the net benefit per appointment. The application of trained models at test time is of course simpler, and integration with administrative systems is here straightforward. Both static and periodically-updated models are feasible without major disruption to current information handling systems in most hospitals. The use of models with probabilistic outputs enables the system to fail gracefully where accuracy might be locally poor for reason of inadequate sampling of the specific neighborhood of predictive features. In such circumstances, detection of high uncertainty can be used to trigger a standard intervention, ensuring that the outcome is no worse than where uniform reminding is used.

Our study makes distinctive contributions to four key aspects of non-attendance predictive modelling.

First, our analysis demonstrates that high-dimensional, high-capacity models of non-attendance are superior to low-dimensional, low-capacity models. Previous studies have either assumed that the problem is tractable within relatively low-dimensional, low-capacity models,^7–9,16,17^ or employed complex modelling without comprehensive evaluation of the relation between performance and complexity.^5,6,17^ Our conclusion suggests the exploration of more complex models of behavior related to scheduled hospital activity is likely to be rewarding.

Second, we demonstrate that state-of-the-art predictive models of non-attendance can be derived from relatively modest datasets, based on routinely recorded, easily-accessible attendance variables, enabling institutions to build effective models without substantial modification of their data streams, and without the necessity—and potential information governance risk—of pooling data across multiple environments.

Third, we provide a formula for calculating the net resource benefit of implementing a predictive model of non-attendance compared with indiscriminate intervention. This quantifies the relation between the model threshold and the resultant net benefit of selective intervention. We use net benefit curves to demonstrate the advantage of deploying predictive modelling across the full plausible range of prevalences of non-attendance and interventional efficacy. Others may employ this approach to construct models that explicitly maximize resource gain, for example by adding a net benefit term to the training loss.

Fourth, we address the problem of class imbalance, neglected in the current literature, quantifying the utility in this task of three methods for imbalance handling: class weights, random undersampling of the majority class, and SMOTE oversampling of the minority class, and providing precision recall curves of performance.

## Methods

### Dataset

A comprehensive, unselected, sequential set of administrative appointment data covering MRI radiological activity at University College Hospital and the National Hospital for Neurology and Neurosurgery was collated for the period between 10th January 2014 and 11th December 2016. The dataset was filtered to include only non-cancelled appointments, yielding 22,318 appointments across 17,295 patients at the two hospital sites. The variables included detailed scheduling data, previous appointment activity, postcode-discretized patient home location, details of MRI scan type and requestor, and aggregate patient costs (Supplementary Table 1). We included all the variables available on our radiology administrative system except those that were empty or redundant. The prevalence of missing values was 4.6%.

The demographic variables of age, sex, ethnicity, employment, or religion were not available within the radiology administrative system from which the data was sourced. We did not seek to obtain these variables from other systems because we wished to determine the performance achievable within the constraints of a routine administrative environment, and were not in a position to determine their marginal value because they cannot be accessed under the information governance framework of the present study. Clinical variables were not modelled for the same reasons, but the clinical diversity and representativeness of the population is conveyed by the distribution of MR imaging study types given in Supplementary Fig. 1.

### Data pre-processing

The dataset was cleaned to remove empty columns or redundant variables. Keyword scan descriptors were extracted from the ‘scan type’ field, and recoded as dummy binary variables.

Further recoding was performed to facilitate modelling: postcodes were converted to longitude and latitude; requesting clinician grades were binned into junior, middle, and senior; and dates were binned into days of the week and month. For the same reason, some implicit associations between variables were made explicit: geodesic travel distance was calculated from home and scan location; referral lag from booking date to appointment date; time since last non-attendance from referral date and last non-imaging non-attendance date. The full list of variables is given in Supplementary Table 1.

Patients attending more than once within the study period provide more information about their attendance than those captured only once. To remove this potential source of bias, the attendance record for each appointment was censored to exclude information on succeeding appointments for the same patient.

Features trivially predictive of the outcome, for example arrival date set at null, were removed from the analysis.

All categorical data was converted to dummy variables, and missing values were imputed as median—except the reciprocal of ‘time since last non-attendance’, which was imputed as 0. The resulting numerical array was transformed into *z*-scores.

### Modelling

We began by modelling all features since no assumptions can be made about the relevance of any specific one. We randomly split the dataset into three stratified subsets: training, validation, and test. Training data was used to derive a set of candidate data-driven models, validation data to optimize the models, and test data to evaluate the top performing model performance and net benefit. These partitions were kept separate; allocation was wholly random with the following ratios: 9:1 training to test, and within the training set, 5:1 training to validation.

To quantify the importance of model complexity and to avoid the risk of methodological over-fitting we constructed and evaluated models based on several standard machine learning architectures: logistic regression, Support Vector Machines (SVM),^18^ Random Forest, AdaBoost,^19^ and Gradient Boosting Machine (GBM).^20^ Each architecture varies in its capacity to handle complex relations between the predictor variables, as discussed below.

In keeping with the broader population, attendances in our dataset outnumbered non-attendances by 10 to 1. Such class imbalance can bias models to the majority class. To counteract this, we separately tested the effect of randomly under-sampling of the majority class, Synthetic Minority Over-sampling Technique (SMOTE) over-sampling of the minority class,^21^ and altering class weights to penalize classification mistakes in the minority class. AdaBoost or GBM models were excluded from this procedure since class imbalance is internally handled by adaptive boosting.

Hyper-parameters were optimized by 10-fold cross-validated grid-search within the training subset (Supplementary Table 2). Average area under the Receiver Operating Characteristic curve (AUC) was used for scoring, a common classification metric that balances sensitivity and specificity.

### Testing

The best candidate model was finally tested on the held-out test set, quantifying performance separately by AUC and by average precision. Average precision is a robust metric in the presence of class imbalance since it excludes the ‘true negatives’ constituent in specificity, focusing instead on precision, or positive predictive value.

### Quantifying the effect of model dimensionality

The relation between the complexity of the model and its performance can be quantified in two ways: first, by the differential performance of model architectures varying in expressive capacity, and second, by creating models based on the best architecture that vary systematically in the number of input features. Here, we used the Gini-importance index from the best all-feature GBM model to rank each feature, and created separate models including features incrementally added in rank order from 1 to 137, evaluating the AUC at each step. Note no grid search was performed, as this would be prohibitively expensive computationally and is unlikely to alter the relative feature rank.

### Impact modelling

The value of a predictive system depends on the relative cost of a lost appointment, and the cost and efficacy of the intervention. The mean ‘reference’ cost of an MRI in the UK National Health Service for the latest available reporting period (2015–2016) is £147.25,^22^ rounded to £150. The cost of reminding a patient by telephone—which often requires more than one call—is conservatively estimated at £6 within our institution, in broad agreement with commercial rates. The reported intervention efficacy ranges from 33 to 39%:^11–13^ here we conservatively choose the lower value.

A set of derived metrics enables us to quantify the value of guided intervention. *Call efficiency*, equal to the positive predictive value, is the ratio of the number of correct interventions to the total number of suggested interventions. The *number needed to call*, the number of telephone calls required to prevent one non-attendance, is the reciprocal of call efficiency. The *net benefit* of using a given predictive model compared with intervening in all appointments, is given by the following equation:

1 $$NB_j = B \cdot TPR_j \cdot P - C \cdot TPR_j \cdot P - C \cdot FPR_j \cdot \left( {1 - P} \right) - \left( {B \cdot P - C} \right)$$

where *NB* is the net benefit, *B* is the average cost saving given the intervention, *TPR* is the true positive rate, *FPR* is the false positive rate, *P* is the prevalence of non-attendance, *C* is the cost of the intervention, and *j* is the test parameter threshold. This allows us to estimate the net benefit across a range of values for *B* and *P*, given reasonable values for *C*, across the full range of *j*. To calculate of net benefit based on current values, we set *B* = 50, *C* = 6, and *P* = 0.09, where *B* is the estimated value of a missed appointment (£150) multiplied by the estimated efficacy of intervention (33%). Our approach here is adapted from the established quantification of net benefit in clinical investigation.^23^

The foregoing refers to operating benefit and excludes the capital cost of building the model and support infrastructure. This will vary with the capabilities of the institution: our own internally estimated one-off cost of ~£20,000 is plausibly representative, with long-term support absorbed into existing analytic resource.

The benefit to an institution as a whole naturally depends on clinical activity. Here, we take as representative the overall outpatient activity of the average UK National Health Service Hospital Trust, estimated at ~800,000 events per year.^1^ The narrower activity related to MRI is estimated at 20,000 annually per hospital trust.^24^

### Analytic environment

All modelling was done in Python 2.7 and using open source packages. Specifically, data pre-processing was conducted with NumPy,^25^ Pandas,^26^ and Scikit-Learn;^27^ geographic calculations with GeographicLib;^28^ and visualizations with Matplotlib.^29^ All models were built using Scikit-Learn. The hardware specification used was: 32 GB memory, Intel® Xeon(R) CPU E5-2620 v4 @ 2.10 GHz × 32 processor, and GeForce GTX 1080/PCIe/SSE2 graphics.

### Ethics

This study was approved by the University College London Hospitals NHS Trust. The study was classified as a service evaluation and optimization project using irrevocably anonymized data, which does not require ethical approval or consent.

## Supplementary information

Supplementary Information

## Data Availability

The minimum dataset required to replicate this study contains personal data and is not publicly available, in keeping with the Data Protection Policy of University College London Hospitals NHS Foundation Trust.

## Supplementary information

**Supplementary information** accompanies the paper on the *npj Digital Medicine* website (10.1038/s41746-019-0103-3).

## Code availability

The code used in this study is currently unavailable but may become available in the future from the authors on reasonable request.
