# Supplementary material for: Predicting scheduled hospital attendance with artificial intelligence
Source: NPJ Digit Med. 2019 Apr 12;2:26. doi: 10.1038/s41746-019-0103-3 (PMC6550247; doi:10.1038/s41746-019-0103-3)
Supplement: Supplementary file 1 — Supplementary Information [file 41746_2019_103_MOESM1_ESM.pdf]

Supplementary Material

Supplementary text, two tables, and three figures:

Systematic review

Table 1. Variable description.

Table 2. Hyperparameter values optimised by grid search.

Figure 1. Distribution of MR study types.

Figure 2. Distribution of attendance.

Figure 3. Comparative model performance.

Systematic review of the literature

We searched Scopus and Google Scholar with the following terms in the indicated logical relation: ("non-attendance" OR "no-show" OR "missed appointment") AND ("prediction" or "modelling" OR "logistic regression" OR "support vector machine" or "random forest" or "neural network" or "machine learning" or "artificial intelligence") AND ("prediction" OR "modelling" or "predictive analytics") AND ("appointment" OR "outpatient" OR "clinic"), for all records until 20 August 2018. Of the 991 records identified, 210 were manually found to be concerned with attendance modelling, and were closely examined to select those including robustly generalisable measures of performance such as out-of-sample prediction. The eight studies satisfying this requirement achieved a median area under the receiver operating curve of 0.7315, employing models with a median of 18 variables.

Variable description	Dummy	Top 81
Appointment day Friday	Y	N
Appointment day Monday	Y	N
Appointment day Saturday	Y	Y
Appointment day Sunday	Y	N
Appointment day Thursday	Y	N
Appointment day Tuesday	Y	N
Appointment day Wednesday	Y	N
Appointment month April	Y	Y
Appointment month August	Y	Y
Appointment month December	Y	Y
Appointment month February	Y	N
Appointment month January	Y	N
Appointment month July	Y	N
Appointment month June	Y	Y
Appointment month March	Y	Y
Appointment month May	Y	N
Appointment month November	Y	Y
Appointment month October	Y	N
Appointment month September	Y	N
Clinic appointment count	N	Y
Days from referral to appointment	N	Y
Direct access appointment count	N	Y
First Appointment count	N	Y
Follow up appointment count	N	Y
Geodesic distance from home address to scan	N	Y
Home address count	N	Y
Imaging appointment count	N	Y
Junior staff appointment count	Y	Y
Middle grade staff appointment count	Y	N
No address listed	N	N
Outpatient appointment count	N	Y
Outpatient procedure appointment count	N	Y
Patient address latitude	N	Y
Patient address longitude	N	Y
Previous imaging DNA count	N	Y
Previous non-imaging DNA count	N	Y
Previous scan count	N	Y
Referral day Friday	Y	N
Referral day Monday	Y	N
Referral day Saturday	Y	N
Referral day Sunday	Y	Y
Referral day Thursday	Y	Y
Referral day Tuesday	Y	Y
Referral day Wednesday	Y	N
Referral month April	Y	Y
Referral month August	Y	Y
Referral month December	Y	Y
Referral month February	Y	N
Referral month January	Y	Y
Referral month July	Y	N
Referral month June	Y	N
Referral month March	Y	Y
Referral month May	Y	Y
Referral month November	Y	N
Referral month October	Y	Y
Referral month September	Y	Y
Scan type abdomen	Y	Y
Scan type adrenals	Y	N
Scan type angiography	Y	N
Scan type ankle	Y	Y
Scan type arm	Y	Y
Scan type brachial	Y	Y
Scan type brain injury	Y	N
Scan type breast	Y	Y
Scan type calves	Y	N
Scan type carotids	Y	Y
Scan type cervical spine	Y	Y
Scan type cervix	Y	N
Scan type cholangiopancreatography	Y	Y
Scan type contrast	Y	Y
Scan type contrast angiography	Y	N
Scan type cranial nerves	Y	Y
Scan type csf flow	Y	N
Scan type defecating proctogram	Y	Y
Scan type diffusion tensor imaging	Y	N
Scan type dissection	Y	N
Scan type elbow	Y	Y
Scan type extracranial	Y	Y
Scan type fistula	Y	N
Scan type foot	Y	Y
Scan type functional	Y	N
Scan type gadolinium	Y	Y
Scan type gamma knife	Y	Y
Scan type hand	Y	Y
Scan type head	Y	Y
Scan type hips	Y	Y
Scan type humerus	Y	Y
Scan type internal auditory meati	Y	Y
Scan type intracranial	Y	N
Scan type knee	Y	Y
Scan type leg	Y	Y
Scan type liver	Y	Y
Scan type lumbar spine	Y	Y
Scan type magnetization transfer	Y	N
Scan type neck	Y	Y
Scan type neuronavigation	Y	N
Scan type orbits	Y	N
Scan type other	Y	N
Scan type parotid	Y	N
Scan type pelvis	Y	Y
Scan type penis	Y	Y
Scan type perfusion	Y	N
Scan type pituitary	Y	Y
Scan type plexus	Y	N
Scan type prostate	Y	Y
Scan type rectum	Y	N
Scan type renal	Y	N
Scan type sacroiliac joint	Y	Y
Scan type sacrum	Y	N
Scan type sedation	Y	N
Scan type shoulder	Y	Y
Scan type small bowel	Y	Y
Scan type spectroscopy	Y	N
Scan type spinal cord	Y	N
Scan type stealth	Y	N
Scan type temporal lobe	Y	Y
Scan type temporomandibular joint	Y	N
Scan type thighs	Y	N
Scan type thoracic spine	Y	Y
Scan type thorax	Y	Y
Scan type thymus	Y	N
Scan type time of flight	Y	Y
Scan type trigeminal nerve	Y	Y
Scan type venogram	Y	N
Scan type venography other	Y	N
Scan type volume	Y	N
Scan type whole body	Y	Y
Scan type whole spine	Y	Y
Scan type wrist	Y	Y
Scanner latitude	N	N
Scanner longitude	N	N
Senior staff appointment count	Y	Y
Telephone appointment count	N	Y
Total cost of patient appointments	N	Y
Unique consultant count	N	Y
Unique specialties count	N	Y
Unique subspecialties count	N	Y

Supplementary Table 1. Variable description.

Model	Parameter	Values
**Gradient Boosting Machine**	loss<br>learning_rate<br>n_estimators<br>max_depth<br>min_samples_split<br>min_samples_leaf<br>max_features<br>subsample	Deviance, **exponential**<br>1, **0.1**, 0.01, 0.001, 0.0001<br>50, 100, 200, 300, 400, **500,** 550, 600<br>1, 2, **3**, 5, 8<br>2, **4**, 10<br>**1**, 5, 10<br>**None**, auto<br>**1**, 0.9
**AdaBoost**	Decision tree max_depth<br>Decision tree min_samples_leaf<br>n_estimators<br>learning_rate	**1**, 2, 5, 8, 10, 15<br>2, 3, 5, 10, **20**, 40<br>100, 250, 300, **350**, 400, 450, 500<br>0.6, **0.7**, 0.8, 0.9, 1, 1.1
**Random Forest**	n_estimators<br>max_depth<br>min_samples_split	100, 200, 250, 300, **360**, 400, 450, 500<br>1, 2, 4, 5, 8, **10**, 30, 40, 50<br>2, 5, 10, 20, **50,** 70, 80, 90, 100
**Support Vector Machine**	Kernel<br>C<br>Gamma (rbf kernel only)	Linear, **Rbf**<br>0.01, 1, **10**, 100, 1000<br>1, 0.1, **0.01**, 0.001, 0.0001
**Logistic Regression**	penalty<br>C<br>Tol	**L1,** L2<br>100, 10, 1, 0.1, 0.01<br>0.01, 0.001, 0.0001, **0.00001,** 0.000001

Supplementary Table 2. Hyperparameter values optimised by grid search. The values found to be optimal are highlighted in bold. A full description of the parameters is available in Scikit Learn documentation.

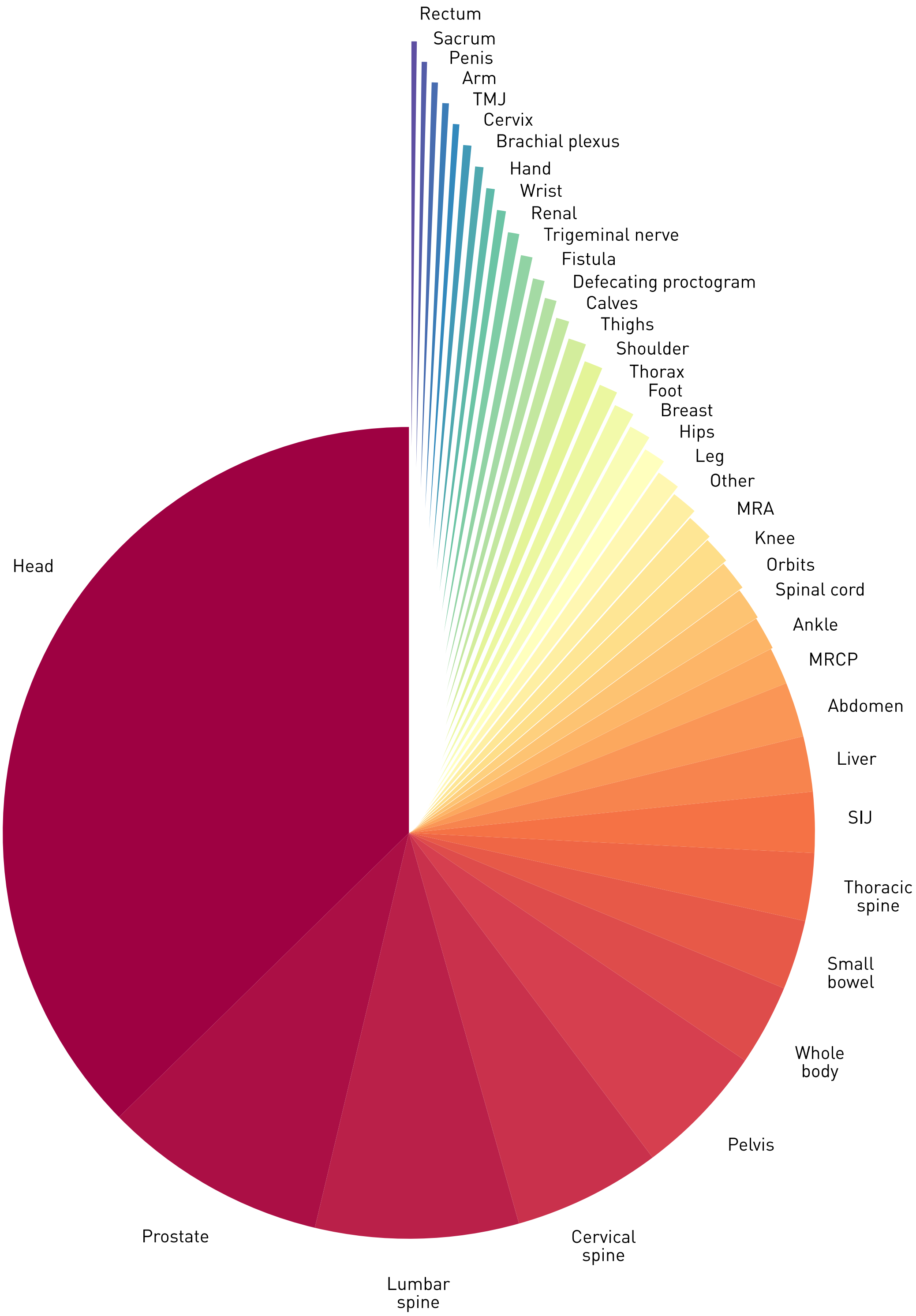

Supplementary Figure 1. Distribution of MR study types.

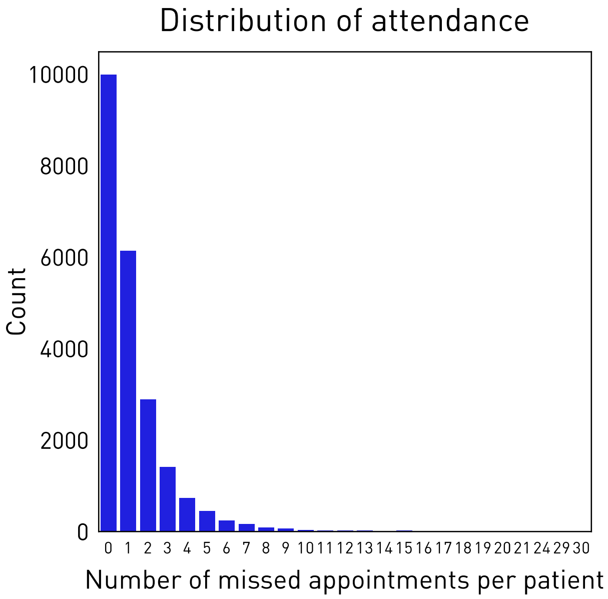

Supplementary Figure 2. Distribution of attendance in our sample.

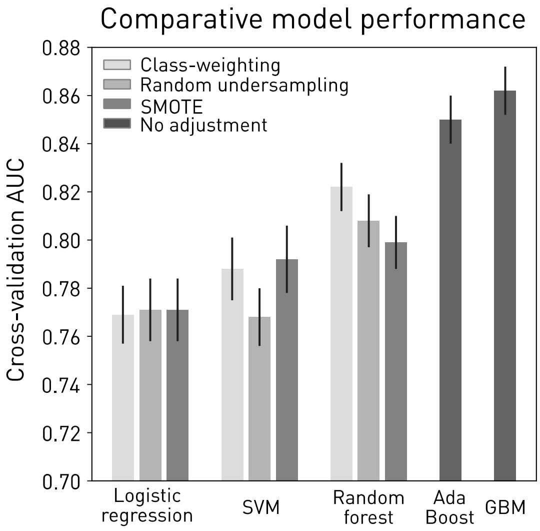

Supplementary Figure 3. Classification performance by model type and imbalance correction, as measured by the area under the receiver operating curve on the training data. Performance improves broadly with increasing complexity of model; class weights perform better than SMOTE or random under-sampling techniques.
